# Injury-related deaths in Enugu, Nigeria from 2010 to 2016: a descriptive review

**DOI:** 10.11604/pamj.2020.36.266.25273

**Published:** 2020-08-11

**Authors:** Samuel Robsam Ohayi, Nnaemeka Thaddeus Onyishi, Mark Sunday Ezeme

**Affiliations:** 1 Department of Pathology, Enugu State University Teaching Hospital and College of Medicine, Park Lane, Enugu, Nigeria,; 2 Department of Psychiatry, Enugu State University Teaching Hospital and College of Medicine, Park Lane, Enugu, Nigeria

**Keywords:** Injury-related, death, coroner, public health, descriptive, homicide, accidents

## Abstract

**Introduction:**

death from injuries is a global public health problem. Ninety percent occur in low- and middle-income countries like Nigeria. This study aimed to determine the burden and demographic characteristics of injury-related death in Enugu, Nigeria.

**Methods:**

this is a retrospective study of injury-related deaths in Enugu over a 7-year period. Standardized forms were used to collect data from autopsy reports archived in the Forensic Unit of Enugu State University Teaching Hospital, Enugu and a descriptive analysis of collected data performed.

**Results:**

of the Coroner deaths examined in the period reviewed, 1,067 (86.9%) were injury-related. The male-to-female ratio was 5.2: 1. Mean age of victims was 34.2 ± 14.3years and range was 8 months to 86 years. Most victims (56.7%) aged 21-40 years. Accidents accounted for most deaths (53.2%) followed by homicide (44.3%). Road traffic deaths (51.4%), cult/gang violence (20.8%) and robbery (14.7%) were the commonest. Suicide (0.5%) and domestic violence (0.7%) were the least. More females died in domestic incidents while more males died in all other circumstances. Firearm (56.7%) was the most common weapon followed by knife (19%). Knife and wood (28.7% each) were the commonest weapons in domestic violence. Generally, fatal incidents occurred more in the day-time (65.5%). Most robberies (80.4%) occurred at night. Most cult/gang killings (75.2%) and robberies (81.7%) occurred in public places and at homes respectively.

**Conclusion:**

injury is the highest source of Coroner's death in Enugu. Efforts to curb it are insufficient. A definitive policy on the prevention and management of injury-related deaths is needed.

## Introduction

Injury-related death is a major global public health problem especially in developing countries. A global burden of disease study showed that injuries accounted for 11% and 8% of the total burden of disease in low- and middle-income countries (LMIC) respectively [1]. About 9% of deaths worldwide arise from such injuries and 90% of those deaths occur in low- and middle-income countries including Nigeria [2]. Injury-related death is reportedly on the decline in developed countries [3, 4]. In contrast, death from trauma from various incidents including accidents, interpersonal violence, religious and ethnic conflicts and legal intervention (by law enforcement and other persons with legal authority to use deadly force) is on the rise in developing countries including Nigeria [5, 6]. Active injury surveillance however is lacking [7, 8]. Various factors are known to increase the risk of dying from injuries. Late referral of victims of trauma or non-availability of facilities specialized for the care of serious injuries increase the risk of death from trauma [9]. Being a young male, low socioeconomic status and use of alcohol and other psychoactive substances are associated with increased risk for injury-related death [10]. Rapid rate of urbanisation as is seen in sub-Saharan Africa (SSA) is known to increase risk of death from trauma as it is known to bring about poor city planning and inadequacy of good roads in the face of increasing traffic volume. It is also leads to a population that outgrows economic opportunities with consequent poverty and slums; lack of proper law enforcement and easy availability of illicit brews and drugs [11, 12]. Additionally, poor housing safety measures and poor workplace facilities lead to increased risk of fatal injuries. The cause of fatal injuries depends on the context in which a people live. According to WHO report, homicide rate was nearly three times greater than suicide rate in the African region a trend that differs from the European, Southeast Asian and Western Pacific regions [13]. Homicide is commoner in the urban than rural areas [14]. It has been reported that 90% of all road traffic accident (RTA) deaths occur in low and medium-income countries [15]. Even with these troubling statistics, there is limited data and few studies on trauma fatalities in the LMIC and Nigeria in particular [16]. Also, little attention has been given to this growing epidemic in the developing countries in terms of policy or public health interventions. Given the burden of trauma fatalities in SSA including Nigeria and other developing countries, there is a need for epidemiological research into the characteristics of violent deaths in these areas so as to help develop and improve prevention strategies. The aim of this study is to analyse and determine the burden and demographic characteristics of trauma-related deaths occurring between 2010 and 2016 in Enugu using available post-mortem data from Enugu State University of Science and Technology (ESUT) Teaching Hospital.

## Methods

This is retrospective study which covered a 7-year period. Standardized forms were used to extract data from police records and autopsy reports archived in the Forensic unit of the Department of Histopathology, Enugu State University Teaching Hospital, Enugu and a descriptive analysis of collected data performed. In compliance with the state’s Coroner law, Coroner’s ordinance Cap. 41, the police upon receiving a report about a death occurring under questionable circumstances including injury-related deaths reports such death to a state appointed Coroner (via form B of the Coroner’s ordinance Cap. 14) who then authorises the Pathologist in the Forensic unit (via form C of the Coroner’s ordinance Cap. 41) to perform an autopsy on the body of the deceased. The bodies by statute are delivered to the unit pathologists by the police through the instrument of Coroner forms. These forms are completed in duplicates, each copy endorsed by the Coroner and both submitted to the pathologist. This system therefore centralises post-mortem examination of victims of trauma fatalities from every part of Enugu state in the Forensic unit of the Department of Histopathology of the state university teaching hospital. The pathologist visits mortuaries in different parts of the state where these bodies are deposited accompanied by the investigating police officer who with a relation of the deceased when known and available identifies the bodies to him after which he (the pathologist) carries out a post-mortem examination of the body.

Injury in the context of this study is taken to mean any physical harm on a person's body caused by physical trauma. Only deaths from trauma-related causes that were reported to the Coroner, whether the deceased was brought into the hospital alive or dead, were included in the study. Coroner deaths deemed not to have resulted from trauma were excluded from the study. So also were deaths from legal use of force and deaths in custody. A varied scope of post mortem examination was performed per trauma-death victim and an autopsy report issued to the police via the original copy of the Coroner's form. A duplicate copy of the report which contains all information as the original is archived in the Forensic unit of Histopathology department of ESUT Teaching Hospital, Enugu. Cause of death was categorized by mechanism and intention using the International Classification of Diseases (ICD)-10 codes [17]. The defined injury mechanisms were accident (road traffic, work-related and domestic), suffocation, hanging, drowning, firearms and assorted weapons. Intention for killing a victim was categorized as unintentional (accidental), suicide or homicide. Data were collected by the first author who was assisted by a house officer and a secretarial staff in the department both of whom had been trained for the purpose. Data collected included demographic characteristics of the victim, place, month and time of injury and death, mechanism, nature and site of injury, anatomical cause of death and manner of death. Data were analyzed by simple statistical methods for means and proportions using Microsoft excel. Missing data were small and therefore excluded from the study. Ethical clearance was obtained from ESUT Teaching Hospital’s Ethical committee.

## Results

**Figure 1:**
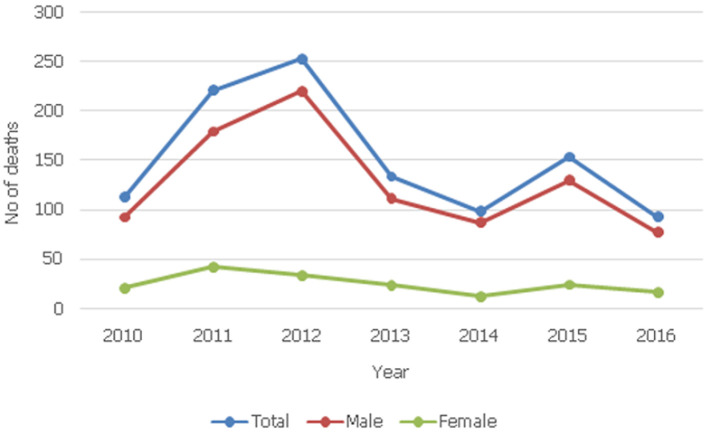
the annual trend of deaths seen from 2010 to 2016

**Figure 2:**
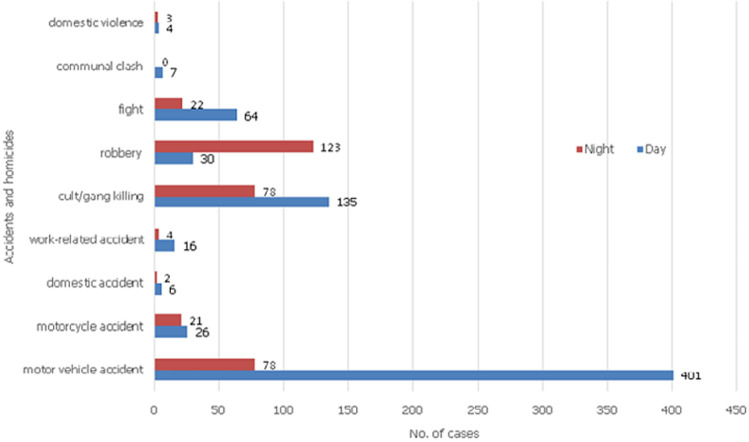
bar chart comparing the time of day in which homicide and accident deaths occurred

A total of 1,228 Coroner deaths were examined in the period reviewed with 1,067 (86.9%) being injury-related and therefore constituted the study population. The male-to-female ratio was 5.2: 1. Mean age of victims was 34.2 ± 14.3 years while the range was 8 months to 86 years. Most victims (56.7%) were aged 21-40 years while those aged ≤ 10 years (4.2%) and > 60 years (6.7%) were the fewest (Table 1). Table 2 shows the age and sex distribution of trauma fatalities against circumstances of the trauma in the study period (n = 1046). Accidents accounted for 568 deaths which is 54.3% of deaths with type of trauma leading to death stated. This was followed by homicide 473(45.2%) and suicide 5(0.5%). The source of trauma in 21 (2% of 1067) cases was unspecified. Road traffic accident death (51.4%), cult/gang violence (20.8%) and robbery (14.7%) were the commonest trauma-causing death in this study. Suicide (0.5%) and domestic violence (0.7%) were the least. A total of 15 deaths (1.43%) occurred from domestic incidents namely accidents, 0.76% and violence, 0.67%. Victims of these deaths were mostly females (66.7%) while in all other circumstances, there were more male victims than females. Work-related accidents accounted for 22 deaths (2.1%). Persons aged 21-40 years made up 65.8% and 46.7% of homicide and accident victims respectively. Firearm (56.66%) was the most common weapon followed by knife (19.03%) as shown in Table 3. Most cult/gang killings (68.8%) and robbery (74%) were by firearms. Knife and wood (28.7% each) were the commonest weapons in domestic violence. Two domestic accidents involved firearms use by children. All suicides were by hanging. Figure 1 shows yearly trend while Figure 2 shows day/night trend of injury fatalities. Fatal incidents generally occurred more in the day-time (65.5%) including road traffic deaths (81.2%) and cult/gang related deaths (63.4%). Most robbery incidents (80.4%) and suicides (60%) occurred at night. Most cult/gang killings (75.2%) and robbery (81.7%) occurred in public places and at homes respectively. No death from accident has been/is being tried in court because perpetrators either escaped arrest or agreed with family of victims for some form of out of court settlement with the permission of the state. Only 8.2% of homicide cases are being tried in court mostly because most perpetrators escaped arrest.

**Table 1:** age and sex distribution of 1067 injury fatalities victims from 2010 to 2016

Year	Sex (n=1067)			Age group in years (n=1054)							
	Male (%)	Female (%)	Total (%)	10 (%)	11-20 (%)	21-30 (%)	31-40 (%)	41-50 (%)	51-60 (%)		Total (%)
2010	92 (8.6)	21(2)	**113 (10.6)**	4 (0.4)	13 (1.2)	43 (4.1)	29 (2.8)	11 (1.0)	8 (0.8)	5 (0.5)	**113 (10.8)**
2011	179 (16.8)	42 (3.9)	**221 (20.7)**	11 (1.0)	20 (1.9)	62(5.9)	51 (4.8)	52 (4.9)	10 (0.9)	11 (1.0)	**217 (20.6)**
2012	220 (20.6)	33 (3.1)	**253 (23.7)**	5 (0.5)	19 (1.8)	96 (9.1)	54 (5.1)	38 (3.6)	24 (2.3)	14 (1.3)	**250 (23.7)**
2013	111 (10.4)	23 (2.2)	**134 (12.6)**	8 (0.8)	12 (1.1)	41 (3.9)	27 (2.6)	16 (1.5)	14 (1.3)	16 (1.5)	**134 (12.7)**
2014	87 (8.2)	12 (1.1)	**99 (9.3)**	3 (0.3)	5 (0.5)	31 (2.9)	26 (2.5)	16 (1.5)	9 (0.9)	9 (0.9)	**99 (9.4)**
2015	130 (12.2)	24 (2.2)	**154 (14.4)**	7 (0.7)	11 (1.0)	46 (4.4)	39 (3.7)	18 (1.7)	15 (1.4)	12 (1.1)	**148 (14.0)**
2016	77 (7.2)	16 (1.5)	**93 (8.7)**	6 (0.6)	7 (0.7)	33 (3.1)	20 (1.9)	12 (1.1)	11 (1.0)	4 (0.4)	**93 (8.8)**
**Total**	**896 (84.0)**	**171(16.0)**	**1067 (100)**	**44 (4.3)**	**87(8.2)**	**352 (33.4)**	**246 (23.4)**	**163 (15.4)**	**91 (8.6)**	**71 (6.7)**	**1054 (100)**

Number of victims whose age was not specified: 13

**Table 2:** age and sex distribution of cases against circumstances of injury fatalities from 2010 to 2016 (n = 1046)

Age group (years)		Accident			Homicide					Suicide (%)	Total (%)
		RTA (%)	Domestic (%)	Work-related (%)							
					Cult/Gang killing (%)	Robbery (%)	Fight (%)	Communal clash (%)	Domestic (%)		
10		41 (3.9)	2 (0.2)	-	-	-	1 (0.1)	-	-	-	44 (4.2)
11-20		47 (4.5)	-	1 (0.1)	19 (1.8)	1 (0.1)	17 (1.6)	-	-	-	85 (8.1)
21-30		166 (15.9)	2 (0.2)	5 (0.5)	101 (9.7)	43 (4.1)	29 (2.8)	-	-	1 (0.1)	347 (33.2)
31-40		99 (9.4)	1 (0.1)	8 (0.7)	59 (5.6)	50 (4.7)	24 (2.3)	3 (0.3)	2 (0.2)	-	246 (23.5)
41-50		91 (8.7)	1 (0.1)	5 (0.5)	20 (1.9)	23 (2.2)	13 (1.2)	2 (0.2)	3 (0.3)	2 (0.2)	160 (15.3)
51-60		42 (4.0)	-	2 (0.2)	13 (1.2)	26 (2.5)	1 (0.1)	1 (0.1)	2 (0.2)	-	87 (8.3)
		44 (4.2)	2 (0.2)	-	6 (0.6)	10 (1.0)	2 (0.2)	1 (0.1)	-	1 (0.1)	66 (6.3)
Unspecified		8 (0.8)	-	1 (0.1)	-	1 (0.1)	-	-	-	1 (0.1)	11 (1.1)
**Total**		**538 (51.4)**	**8 (0.8)**	**22 (2.1)**	**218 (20.8)**	**154 (14.7)**	**87 (8.3)**	**7 (0.7)**	**7 (0.7)**	**5 (0.5)**	**1046 (100)**
Sex	Male (%)	426 (40.7)	3 (0.3)	19 (1.8)	213 (20.3)	128 (12.2)	78 (7.5)	7 (0.7)	2 (0.2)	5 (0.5)	881 (84.2)
	Female (%)	112 (10.7)	5 (0.5)	3 (0.3)	5 (0.5)	26 (2.5)	9 (0.8)	-	5 (0.5)	-	165 (15.8)

Number of fatalities from unspecified circumstances: 21

**Table 3:** distribution of circumstances and weapons/instrument of homicide by year (n = 473)

	Circumstance of death					Total	Homicide Weapon					
Year	Cult/Gang killing (%)	Robbery (%)	Fight (%)	Communal clash (%)	Domestic violence (%)		Firearm (%)	Knife (%)	Piece of wood (%)	Body part/s (%)	Piece of metal (%)	Others^*^ (%)
2010	20 (4.23)	18 (3.81)	9 (1.9)	-	-	**47 (9.94)**	22 (4.65)	10 (2.11)	7 (1.48)	1 (0.21)	6 (1.27)	1 (0.21)
2011	33 (6.98)	14 (2.96)	15 (3.17)	2 (0.42)	1 (0.21)	**65 (13.74)**	29 (6.13)	19 (4.02)	4 (0.84)	-	8 (1.69)	5 (1.06)
2012	52 (10.99)	19 (4.02)	13 (2.75)	-	-	**84 (17.76)**	55 (11.63)	11 (2.33)	8 (1.69)	1 (0.21)	7 (1.48)	2 (0.42)
2013	32 (6.76)	29 (6.13)	6 (1.27)	4 (0.85)	2 (0.42)	**73 (15.43)**	44 (9.3)	17 (3.59)	5 (1.06)	-	3 (0.63)	4 (0.85)
2014	23 (4.86)	27 (5.71)	15 (3.17)	-	1 (0.21)	**66 (13.95)**	36 (7.61)	9 (1.9)	8 (1.69)	5 (1.06)	4 (0.85)	4 (0.84)
2015	39 (8.25)	22 (4.65)	20 (4.23)	-	3 (0.63)	**84 (17.76)**	45 (9.51)	16 (3.38)	10 (2.11)	2 (0.42)	7 (1.48)	4 (0.85)
2016	19 (4.02)	25 (5.29)	9 (1.9)	1 (0.21)	-	**54 (11.42)**	37 (7.82)	8 (1.69)	5 (1.06)	-	3 (0.63)	1 (0.21)
**Total**	218 (46.09)	154 (32.57)	87 (18.39)	7 (1.48)	7 (1.47)	**473 (100)**	268 (56.66)	90 (19.03)	47 (9.94)	9 (1.9)	38 (8.03)	21 (4.44)

* Include fire, nylon rope, electric cable, acid attack, broken bottle, water/drowning etc.

## Discussion

Monitoring for patterns, rates and causes of death is an essential ingredient of health surveillance in a population. Data so generated can inform decision making and resource allocation in health management. Unfortunately, majority of African countries have low quality or no vital registration data [16]. We present a descriptive hospital-based study which gives a snapshot of injury or trauma-related deaths in Enugu Southeastern Nigeria, aiming to describe the distinctive characteristics of the phenomenon. In the absence of population wide data, hospital-based studies, such as this, give some indication of trend and direction of mortality figures in a population. There is high rate of injury mortality in our environment as shown by our study. Our result showed that 84% (896/1067) of the injury deaths occurred in males. By age group, 56.8% (598/1054) of all injury deaths occurred in persons aged between 21 years and 40 years. This pattern of age and sex distribution appears to be a typical characteristic of injury deaths globally as shown by other reports [6, 18, 19]. Males are more affected most likely because by nature they are impulsive, inquisitive and adventurous and therefore engage in risky activities [6]. Seleye-Fubara *et al*. [20] reported that people of this age group are those that mostly engage in the violent militancy in the Niger Delta region of Nigeria. By extension, our finding that victims of deaths from domestic incidents were mostly females (66.7%) tends to reinforce the notion that females are still mostly engaged around the home in our environment. The concentration of injury-deaths in the age segment below population life expectancy makes injury an important cause of premature death. This calls for preventive measures. Also, since that age group is the most productive group of human population such deaths lead to significant economic loss to the society [21-23]. In fact, road traffic accident is among the ten leading causes of total years of life lost (YLL) according to 2016 global burden of disease study [16].

The ICD classification for injuries has two essential aspects namely: the mechanism of the injury (traffic accident, poisoning, suffocation, falling, drowning, fire, firearms etc) and the manner or intent (accidental or unintentional injury, homicide/assault, suicide/self-harm, legal intervention) [17, 18]. By manner or intent, we found accident or unintentional injuries to be the leading cause of injury death, contributing 54.3% (568/1046) of injury deaths. It is followed by homicide which caused 45.2% of injury deaths (473/1046) while only 0.5% (5/1067) was from suicide. This is largely in keeping with global trends. The global burden of disease study 2016 and America's national vital statistic report respectively established unintentional (accidents) injuries as the cause of 70 and 68 percent of all injury deaths [16, 18]. It however contrasts with other studies [20, 24, 25] which reported homicide as the leading cause of injury deaths. Our finding of suicide rate of 0.5% is also markedly different from 18% and 21% reported by other larger studies [16, 18]. Our low suicide rate may be explained by prevailing cultural norms which make suicide a taboo in our society so that suicide deaths are less likely to be reported to the Coroner. Of the 473 homicides in this study, 57% (268/473) and 19% (90/473) were perpetrated with firearms and knife respectively. Most (98.5%) of the firearm homicides were cases of gang killing or robbery. This indicates that while law abiding citizens are precluded from gun ownership by law, criminal elements have managed to acquire guns which are often used in violence. However, with two incidents of accidental firearm deaths caused by children playing with a parent's gun recorded in this study, the dangers of liberal access to guns is highlighted. Measures aimed at controlling the availability of weapons to non-law enforcement personnel will reduce homicide in our setting. The pattern of weapon deployment in domestic violence is that perpetrators mostly used the object that came handy to them in the course of a quarrel suggesting that there killing may not have been a premeditated action.

A careful review of the mechanism and manner/intent of trauma fatalities from this study gives some idea for the reason for the high mortality. That 81.2% RTA deaths occurred in the day suggests that driver error may be part of factors that lead to road accidents. By time trend the spike in trauma deaths in the election years of 2011 and 2015 and the post-election year of 2012 especially in deaths following gang/cult activities may be a fall out of our electioneering activities which are usually characterised by marked intolerance. There is need for further studies into election-related violence in our country as this will help in proper planning for this essential quadrennial exercise in our national life. In the same vein, our findings that 63.4% of cult/gang killings occurred in the day-time and 75.2% of them and 81.7% of robbery-related deaths occurring in public places suggests that perpetrators are very bold and powerful or that the security agencies may not be properly positioned to secure the populace or both. Work-related deaths and deaths from domestic incidents are common according to our findings. This calls for improved safety conditions and appropriate legislation for the enforcement of safety regulations in such places. In addition, it also brings to the fore the issue of workman compensations for work-related morbidities and death. Relations of victims of work-related deaths often narrated their ordeals as they sought for compensations for the death of their relatives. Limitations of this study include underreporting of trauma fatalities, lack of psychological autopsy for suicide cases, lack of death registration by the state and poor mortuary data. Also, the injury deaths analysed in this study are limited to those that came to the attention of the Coroner. While available data might not have given a population-based picture of injury-related deaths in our environment, it has highlighted the attributes of injury-related deaths in a region with data paucity and low reportage.

## Conclusion

Injury is the highest source of Coroner’s death in Enugu with highest contributors being RTA and homicides. Most victims are young adult males. Efforts to curb it are insufficient. There is need for nationwide survey of injury-related fatalities, proper death registration and definitive policy on the prevention and management of injury-related deaths. Specifically, measures should be put in place to remove illegal firearms from the community while also ensuring that new ones do not get in. Road user behaviour as well as the quality of roads and vehicles that ply them needs proper monitoring.

### What is known about this topic

- Trauma is a major contributor to death worldwide;
- An overwhelming majority of such deaths occur in low- and middle-income countries like Nigeria;
- Majority of such deaths is due to road traffic accidents and homicide.

### What this study adds

- Homicide rate is high in our society and they are also carried out in a brazen manner;
- There are numerous unaccounted firearms in circulation in our society, a place whose law enforcement agents are ill-equipped to fight crime;
- Victims of trauma fatalities and or their survivors do not receive appropriate attention.
